# Caries Trajectories From Childhood to Adulthood Associated With Mental Disorders in Midlife

**DOI:** 10.1111/jphd.12665

**Published:** 2025-02-24

**Authors:** Begoña Ruiz, Jonathan M. Broadbent, W. Murray Thomson, Sandhya Ramrakha, Joseph Boden, Geri McLeod, Terrie E. Moffitt, Avshalom Caspi, Richie Poulton

**Affiliations:** 1 Faculty of Dentistry, Universidad de Chile, Santiago, Chile; 2 Sir John Walsh Research Institute, Department of Oral Sciences, Faculty of Dentistry, University of Otago, Dunedin, New Zealand; 3 Dunedin Multidisciplinary Health and Development Research Unit, Department of Psychology, Division of Sciences, University of Otago, Dunedin, New Zealand; 4 Christchurch Health and Development Study, Department of Psychological Medicine, University of Otago, Christchurch, New Zealand; 5 Department of Psychology and Neuroscience, Duke University, Durham, North Carolina, USA; 6 Department of Psychiatry and Behavioral Sciences, Duke University Medical Center, Durham, North Carolina, USA; 7 Center for Genomic and Computational Biology, Duke University, Durham, North Carolina, USA; 8 Social Genetic and Developmental Psychiatry Centre, Institute of Psychiatry, Kings College London, London, UK

**Keywords:** cohort studies, dental caries, dental public health, epidemiology, mental disorders, oral health, public health

## Abstract

Oral and mental health are integral to overall health and well-being. Primary dentition caries is associated with poorer oral and general health and faster aging by midlife, but it remains unclear whether these associations also hold for mental health.

**Objectives:**

To investigate whether primary dentition caries, caries experienced in adolescence and adulthood, and caries trajectories across the life course are associated with mental disorders in the fifth decade of life in two New Zealand birth cohorts.

**Methods:**

Data on childhood caries and adult mental disorders were obtained from the Dunedin Multidisciplinary Health and Development Study and the Christchurch Health and Development Study. Generalized Linear Models (GLMs) were used to estimate associations between caries at age 5 and mental disorders at age 45/40 for Dunedin and Christchurch studies, respectively. Additional analyses using Dunedin Study data investigated associations between permanent dentition caries trajectories from ages 9 to 45 and mental disorders at age 45. All analyses adjusted for sex, perinatal health, childhood SES, childhood IQ, and adult personality.

**Results:**

Primary dentition caries experience was not associated with mental disorders in midlife in either cohort. Dunedin Study participants who were in a less favorable permanent dentition caries trajectory had higher rates of mental disorders at age 45 than those in the low-caries trajectory.

**Conclusions:**

People who experience poor oral health across the life course are also those who suffer from poorer mental health in mid-adulthood. A lifelong trajectory of poorer dental health indicates poorer mental health and well-being in adult life.

## 1 | Introduction

Oral conditions and mental disorders (i.e., disturbances in thought, behavior, and emotion that interfere with or limit social, family, educational, or work activities) [1] are a function of biology, behavior, and socio-political contexts; hence, they are both highly influenced by the living environment. They share common risk factors—with each other and with other noncommunicable diseases (NCDs)—including low socioeconomic status (SES), smoking, alcohol use and stress, and unhealthy diet [2, 3]. Poverty, inequality, and exposure to early childhood adversity (including maltreatment and neglect) are circumstances that might place individuals at a higher risk of experiencing oral and mental ill-health [4].

The associations between oral conditions—like dental caries, periodontal disease, and dental erosion—and mental disorders have been observed in both directions. On the one hand, individuals who experience mental disorders are at higher risk for dental caries, tooth loss, and periodontal disease than those who are mentally healthy [5]. Adults with depression have more untreated caries [6], missing teeth [7, 8], and are less likely to use dental services [9] than their healthy peers. These associations might be a consequence of poor self-care, higher consumption of sugary foods/drinks, comorbidities with tobacco/alcohol use, iatrogenic effects of some medications [3], or barriers to accessing dental care due to affordability or fears [3, 8]. On the other hand, oral conditions and their physical, psychological, and social impacts can lead to lower self-esteem and self-confidence, social isolation, loneliness, and might aggravate or perpetuate mental disorders [3], and may be exacerbated by dental anxiety and poor oral health-related quality of life [10].

The first years of life are crucial to an individual’s development because many early life experiences impact growth and future health. Various childhood factors, including socioeconomic disadvantage [11], adverse childhood experiences (ACEs) [12], poor self-control [13], and low IQ [14], are associated with poor mental and/or physical outcomes in later life. Associations between childhood caries and health outcomes in midlife have been observed across the subjective and physical domains among adults. Primary dentition caries experience was associated with poorer oral health [15], self-reported general health [16], and physical health and aging in midlife [17].

Although associations between oral health and mental health among adult populations have been widely investigated in the past [3, 5, 18, 19], no studies have examined this relationship over the life course. Poor oral health in childhood might also be a risk indicator for future mental ill-health. Here, we extend the reach of previous research by examining longitudinal associations between caries in the primary and permanent dentitions and mental health in midlife.

Accordingly, the aims of this study were to: (1) investigate whether primary dentition caries was associated with mental disorders by age 40–45 years in two New Zealand (NZ) birth cohorts; and (2) examine whether caries experienced in adolescence and adulthood and developmental trajectories of caries experience from ages 9 to 45 years were associated with mental disorders in midlife.

## 2 | Methods

### 2.1 | Study Design and Population

Participants were members of two NZ birth cohort studies: the Dunedin Multidisciplinary Health and Development Study and the Christchurch Health and Development Study (hereafter the Dunedin and Christchurch studies, respectively). Both studies have collected data from several sources on a wide range of domains, including oral health, general health, and mental health and well-being.

The Dunedin Study is a population-representative birth cohort of 1037 individuals (91% of eligible births; 52% boys) born from April 1, 1972 to March 31, 1973 in Dunedin, NZ. Cohort families represent the full SES range of NZ’s South Island, and study participants are primarily of European ethnicity (7.5% self-identify as Māori and 1.5% as Pacific people). Perinatal data were collected at birth, and the cohort for the longitudinal study was defined at age 3 years. The cohort was assessed again at ages 5, 7, 9, 11, 13, 15, 18, 21, 26, 32, 38, and (most recently) at age 45 years, when 938 (94%) of the 997 living cohort members took part. Written informed consent and ethical approval were obtained for each assessment phase.

The Christchurch Study is a birth cohort of 1265 individuals (97% of all births; 50.2% boys) born from April 15 to August 5, 1977 in Christchurch, NZ. Cohort families represent the full SES range of NZ’s South Island. Most participants self-identify as being of European origin, but about 13% report Māori or Pacific ethnicity. The cohort was assessed at birth, 4 months, annually from age 1 to 16 years, and again at ages 18, 21, 25, 30, 35, and 40 years, when 904 (74%) of the 1222 living cohort members participated. Written informed consent and ethical approval were obtained for each assessment phase.

### 2.2 | Primary Dentition Caries and Permanent Dentition Caries Trajectories

Caries experience at age 5 years was assessed according to WHO methods for the Dunedin Study, and collected from routinely collected School Dental Service clinic records at ages 5 or 6 years for participants in the Christchurch study. Caries experience was summarized using the dmft index. For some analyses, participants were categorized into no decayed, missing, or filled teeth (dmft = 0), moderate caries experience (dmft = 1–4), or high caries experience (dmft ≥ 5). Methodological characteristics of oral examinations for both studies have been reported elsewhere [20, 21]. Data were summarized using the dmf index in both studies. In the Dunedin Study, examinations were conducted by four dental examiners, whereas in the Christchurch Study, data were collected from School Dental Service records.

In the Dunedin Study, caries experience was also collected at ages 9, 15, 18, 26, 32, 38, and 45 years. At each age, according to WHO methods, dentists examined teeth for dental caries and restorations, with four surfaces being considered for canines and incisors, and five surfaces for premolars and molars. Caries experience was summarized using the dmf/DMF index at the surface and tooth levels. Through analysis of these data, permanent dentition caries trajectories from ages 9 to 45 were described (*n* = 975) [15]. The six trajectories identified include: “low caries rate”; “moderate caries rate, maintained”; “moderate caries rate, unmaintained”; “high caries rate, restored”; “high caries rate, tooth loss”; and “high caries rate, untreated caries.” For a detailed description, see Table 1 and Supporting Information S1.

### 2.3 | Assessing Mental Disorders in Midlife

In the Dunedin Study, mental disorders were assessed at every assessment phase from age 11 years, and most recently at age 45 years. At age 45 years, psychiatric interviews were conducted by health professionals blind to participants’ prior data, using the Diagnostic Interview Schedule. Participants were asked about past-year symptoms based on the Diagnostic and Statistical Manual of Mental Disorders (DSM) criteria. The following disorders were assessed: internalizing disorders (including major depression, generalized anxiety disorder, fears [such as social phobia, specific/simple phobia, agoraphobia, and panic disorder], posttraumatic stress disorder [PTSD]); externalizing disorders (including attention-deficit/hyperactivity disorder, conduct disorder, alcohol dependence, tobacco dependence, cannabis dependence, and drug dependence); and thought disorders (including obsessive-compulsive disorder [OCD], mania, and schizophrenia). Composite measures of “any internalizing disorder,” “any externalizing disorder,” “any thought disorder,” and “any mental disorder at age 45 years” were computed. Additionally, a measure of “any lifetime mental disorder by age 45 years” (lifetime occurrence) used data on mental disorders from ages 11, 13, 15, 18, 21, 26, 32, 38, and 45 years.

Christchurch Study members completed a detailed mental health interview at age 40 years where they were asked about past-year symptoms of mental and substance use using the Composite International Diagnostic Interview (CIDI). Custom-written survey items were used to assess DSM-5 diagnostic criteria for the following disorders: internalizing disorders (including major depression, generalized anxiety disorder, social phobia, specific/simple phobia, agoraphobia, and panic disorder), PTSD, eating disorders (including bulimia, anorexia and binge eating disorder), and manic episode; and externalizing disorders (including alcohol dependence, cannabis dependence, drug dependence [not cannabis], and tobacco dependence). Composite measures of “any internalizing disorder,” “any externalizing disorder,” and “any mental disorder at age 40 years” were computed. A composite measure of “any lifetime mental disorder by age 40 years” (lifetime occurrence) used data on mental disorders from ages 15–16, 18, 21, 25, 30, 35, and 39–40 years. For details on symptom criteria for both studies, see Supporting Information S1.

### 2.4 | Covariates

The selection of covariates was guided by previous knowledge of some key psychosocial determinants of oral and general health. Sex, perinatal complications, childhood SES, childhood IQ, and personality style in adulthood were included as covariates. The latter was included because personality traits are associated with well-being and psychological health and are relevant to the age 40–45 outcomes. In both studies, family SES was recorded at the child’s birth using the Elley-Irving scale of SES for NZ, which places occupations into six categories ranging from 1 = professional to 6 = unskilled laborer. Perinatal complications were recorded shortly after birth, with Study members classified as 0 with none, or as 1+ where there were ≥ 1 perinatal complications. Childhood IQ assessment used the Wechsler Intelligence Scale for Children–Revised (WISC–R) according to standard protocols. The IQ variables were standardized to population norms (mean = 100, SD = 15) in both studies. Personality style in adulthood in the Dunedin study was assessed by the Multidimensional Personality Questionnaire (MPQ) adapted for NZ, with the three superfactors of negative emotionality, positive emotionality, and constraint. In the Christchurch study, personality was assessed using the “Big-Five” personality dimensions. Neuroticism, extraversion, and conscientiousness were used as analog scales of MPQ superfactors. For these analyses, personality scores were standardized into *Z* scores (mean = 0, SD = 1). For a detailed description of covariates, see the Supporting Information S1.

### 2.5 | Statistical Analysis

Bivariate analyses for mental disorders by age 5/5–6 years caries were conducted separately for each study. Generalized linear models (GLMs) were used to estimate associations between caries at age 5 or 6 years and mental disorders at age 45/40 for the Dunedin and Christchurch cohorts, respectively. GLM models using poisson family, log link function, and robust variance were specified to estimate incidence rate ratios for mental disorders by “any caries experience” (dmft > 0 at age 5). Analyses used Stata/SE 17.0 (StataCorp LLC, College Station, TX, USA). Analyses adjusted for sex, perinatal health, childhood SES, childhood IQ, and adult personality style and were guided by a directed acyclic graph (Figure S1). Data reporting followed STROBE guidelines.

Using Dunedin Study data, we first examined whether participants in the highest quartile for dmfs/DMFS score at each of the ages 5, 9, 15, 18, 26, 32, 38, and 45 years had higher rates of mental disorders at age 45 years than those in the lower quartiles or those who were caries-free; secondly, whether permanent dentition caries trajectories from ages 9 through to 45 years were associated with higher rates of mental disorders at age 45 years. For the latter analyses, the “low caries rate” group was used as the reference category. Again, all analyses adjusted for sex, perinatal health, childhood SES, childhood IQ, and adult personality.

## 3 | Results

For Dunedin Study participants, data on both primary dentition caries and internalizing, externalizing, and thought disorders at age 45 years were available for *n* = 830, *n* = 833, and *n* = 833, respectively. For Christchurch Study participants, data on both primary dentition caries and internalizing and externalizing disorders at age 40 years were available for *n* = 824 and *n* = 820, respectively. Data on caries at age 5 or 6 years and lifetime mental disorders were available for *n* = 912 and *n* = 970 participants in the Dunedin and Christchurch studies, respectively. Past-year prevalence estimates for “any mental disorder” at ages 40 and 45 years were 33.0% and 39.0% for the Christchurch and Dunedin studies, respectively. Past-year prevalence estimates for “any mental disorder from ages 14 to 40 years” in the Christchurch Study was 83.1% and from ages 15–45 years in the Dunedin Study was 85.5%. No evident gradients were observed in the bivariate analyses between primary dentition caries and mental disorders in midlife for either cohort (Table 2).

In the final models, Dunedin Study participants who had experienced caries by age 5 years were more likely to have any of six anxiety disorders or simple phobia at age 45 years than those who were caries-free; however, these findings were not replicated in the Christchurch cohort (Table 3) or when examining associations between categories of caries experience (dmfs/DMFS = 1–4; dmfs/DMFS ≥ 5) and mental disorders (data not shown).

**FIGURE 1 |:**
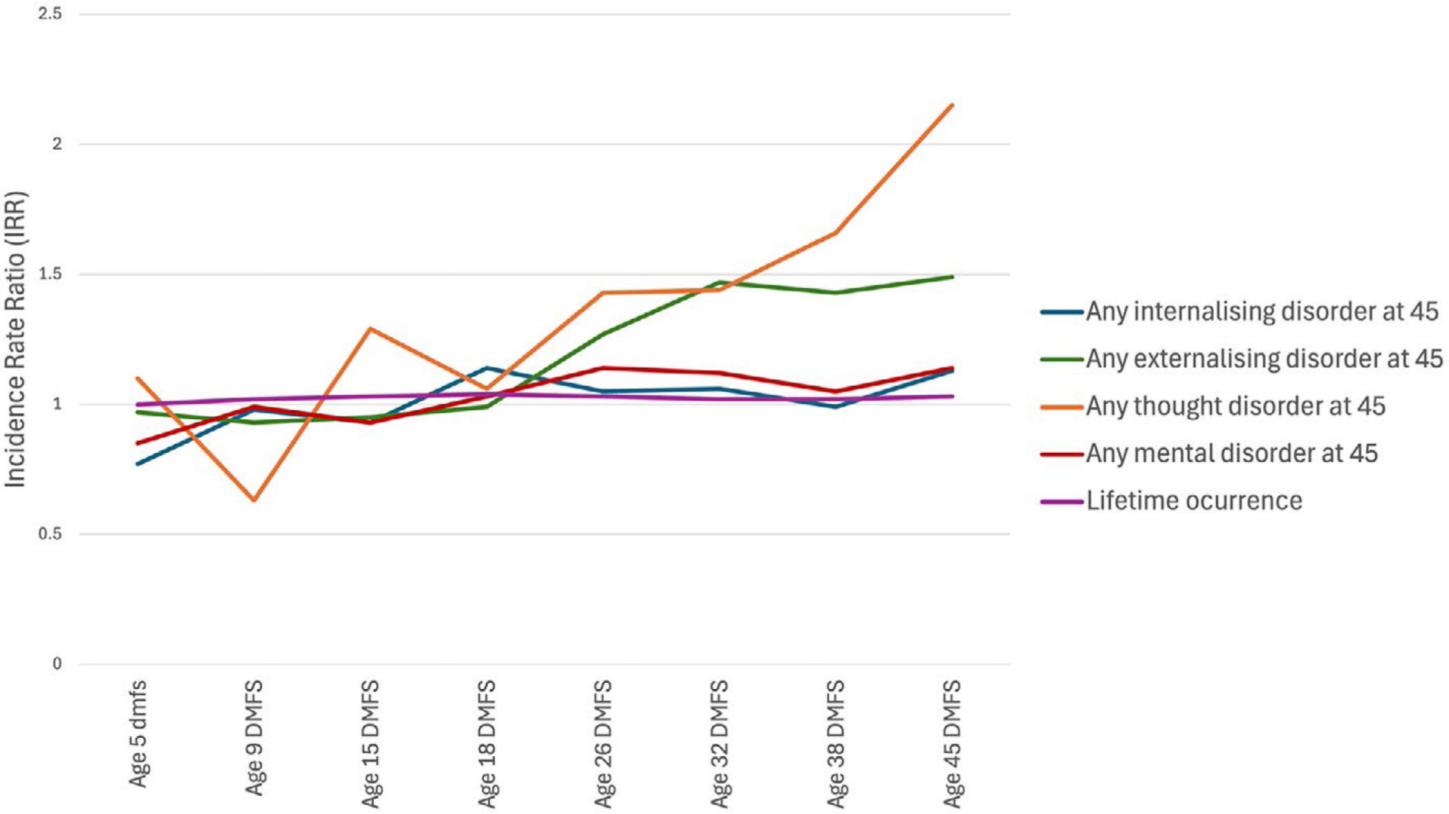
Associations between highest quartile of dmfs/DMFS score at each age (from ages 5 to 45 years) and mental disorders at age 45 years among Dunedin Study participants.

Dunedin participants who were in the highest quartile for caries experience at age 32 years were more likely to have an externalizing disorder at age 45 than those who were in the lower quartile for DMFS or who were caries-free. Consistent associations were observed for those who were in the highest quartiles for caries experience at ages 38 and 45 years (Figure 1). Additionally, those in the highest quartile for DMFS at ages 26, 32, 38, and 45 years were more likely to be tobacco-dependent at age 45 than those who were in the lower quartiles or who were caries-free (Table S1).

Permanent caries trajectories from ages 9 through to 45 years in the Dunedin Study were associated with mental disorders at age 45, whereby participants in the “Moderate rate, unmaintained” and in the “High rate” trajectories (restored, tooth loss, and untreated caries) were more likely to have an externalizing disorder than those who were in the “Low caries rate” trajectory group (Table 4). Those in the high-rate groups—characterized by tooth loss and untreated caries—were also more likely to have a thought disorder by age 45 years. Participants in the “High rate, tooth loss” trajectory group were more likely to have any mental health disorder at age 45 years than those in the low caries rate groups. Associations between the “Moderate rate, unmaintained” and the remaining high-rate groups (high rate, restored and high rate, and untreated caries) and mental disorders at age 45 years were in the same direction of association, but showed smaller effects. Participants in the “Moderate rate” groups and in the “High rate, untreated caries” group were more likely to have had a lifetime diagnosis of any mental health disorder than those who were in the low caries trajectory group.

## 4 | Discussion

This study of two birth cohorts, followed into the fifth decade of life, contributes to advancing knowledge of the oral-mental health relationship in four ways. First, it found primary dentition caries at age 5/5–6 years (as a one-time measure) not to be associated with mental disorders in midlife. Second, Dunedin Study data confirmed cross-sectional associations between participants in the worst quartile for DMFS and higher rates of externalizing disorders and, more specifically, tobacco dependence at age 45 years. Third, from the early 30s, higher caries experience was associated with higher rates of externalizing disorders. Finally, developmental trajectories of permanent caries experience assessed from ages 9 to 45 years were associated with higher rates of mental disorders at age 45 years and with lifetime occurrence of mental disorders.

Primary dentition caries at age 5 years were not longitudinally associated with mental disorders by age 40/45 years. Previous findings from the same two cohorts found caries experience at age 5/5–6 years to be associated with poorer self-reported general health [16] and biomarkers for poor health and aging by midlife [17]. Thus, we hypothesized that childhood caries would also indicate poorer mental health later in life. However, caries at age 5 years—as a single measure—was not associated with poorer mental health by midlife. The prevalence of mental disorders at the ages 40 and 45 years was 33% and 39% for the Christchurch and Dunedin studies, respectively. It may be that associations between childhood caries and adult mental health become evident as study members age and their mental health and well-being concomitantly decline. It could also be argued that it is the more proximal factors or evidence of oral disease surrounding the adolescent or adult developmental epochs that better indicate risk for poorer adult mental health. As previously reported, Dunedin cohort members with enduring positive mental health did not differ in SES background, physical health, or cognitive ability from those who met the diagnostic criteria for a mental disorder [22]. Instead, having an advantageous personality style and no family history of mental disorder characterized the minority group with remarkable mental health. It has been reported that adult experiences are key in determining individuals’ risk of adult depression and anxiety [23]. However, a small proportion of depressed and anxious individuals have childhood risk (family psychiatric history, childhood adversity and behavior, and predisposing personality traits) [24].

Dunedin Study findings showed that having higher caries experience (being in the worst quartile for DMFS) at age 45 years was associated with higher rates of externalizing, thought disorders, and tobacco dependence at age 45 years, showing coexistence of oral and mental health problems in adulthood. Other cross-sectional studies have reported similar associations between caries experience or tooth loss and mental health, but mainly in older adults (50+ years) and focused on depression [25–27]. A recent umbrella review reported associations between caries experience and severe mental illness or substance use [5]. Adults with depressive, anxiety, or eating disorders or who were substance users were more likely to have caries or be edentulous than those who were mentally healthy. However, from an analysis of the data from the early 30s, this study found longitudinal associations between higher caries experience and higher rates of externalizing disorders at age 45 years.

Moreover, enduring poor dental health represented by those participants who followed the less favorable developmental permanent dentition caries trajectories (“high-rate, tooth loss” and “high-rate, untreated caries”) had higher rates of externalizing or thought disorders by age 45 years. Those who have accumulated the highest burden of dental caries—by losing teeth or by having the greatest unmet “restorative” needs—also had poorer mental health in the fifth decade of life. Other longitudinal studies conducted among Japanese [28], English [29], Chinese [30], and American [31] older adults have reported associations between tooth loss, edentulousness, and use of dentures and depressive symptoms. None have examined disorders outside the internalizing family (anxiety/depressive disorders) or used life course caries data. Oral conditions, mental disorders, and other NCDs share common determinants, including low SES, smoking, alcohol use and stress, unhealthy diet, and physical inactivity. Individuals who experience poorer dental health throughout their lives also experience higher rates of multiple mental disorders by midlife. These longitudinal associations have shown that both conditions coexist, and so a better understanding of the psychosocial pathways (i.e., common causes) that connect them may facilitate their management and prevention. Participants’ membership in the worst caries trajectories reflects their experience of a broad range of adversities and supports the idea of orienting health services to aid the proportion of people who have oral health needs but also experience poorer mental health. Additionally, dentists should be aware of patients who suffer from dental anxiety, or phobia, as people with dental anxiety tend to have more caries, missing teeth, poorer oral health-related quality of life (OHR-QoL) [32] and avoid dental care [33]. It is worth investing in oral health in childhood (and throughout the life course), considering that oral health impacts well-being, mental health, and quality of life for individuals across life. We believe that it is the shared social (macrostructural and commercial) determinants between mental disorders and oral conditions, the “causes-of-the-causes” that explain these associations (whereby mechanisms going in both ways involve interconnected social, psychological, and behavioral processes). Global oral health policies and healthcare systems should acknowledge the need to address structural determinants of all chronic conditions. An example of this is the incorporation of sugar control into the World Health Organization’s NCD approach [34].

The strengths of this study include the use of two representative population-based birth cohorts with high retention rates followed up into midlife. Second, standardized clinical dental measures (dmfs/DMFS) were used. Third, mental disorder data did not come from clinical settings but from observations of two general populations. Fourth, all analyses included covariates known to be important determinants of health and well-being. Fifth, personality was included as a covariate because personality traits can affect morbidity and mortality and predict outcomes in middle-age and later-life [35]. Some limitations include: despite the samples being representative, they were both predominantly of European ethnicity, reflecting the NZ South Island’s demographics at the time of study. We acknowledge the limitations of purely observational studies and do not claim causation. The possibility that other known or unknown variables may affect the observed associations should not be dismissed. Caries trajectories were identified only in the Dunedin cohort due to the availability of dental data.

To conclude, primary dentition caries experience at the age of 5/6–6 years was not associated with poorer mental health in mid-life. However, longitudinal associations between higher caries experience and higher rates of externalizing disorders and tobacco dependence at age 45 became evident from the mid-20s. Life course dental data describing permanent dentition caries experience trajectories showed that people who have experienced poor oral health cumulatively across the life course are also those who have higher rates of mental disorders in mid-adulthood. Our findings give support for public health measures aiming to tackle the wider structural and social factors that favor oral health problems in childhood, adolescence, and adulthood, because it is likely that oral and mental health conditions coincide in the same individuals. A lifelong trajectory of poorer dental health also indicates poorer mental health and well-being in adult life.

## Supplementary Material

sup table 1

sup table 2

sup table 3

sup table 4

Supporting Information

Additional supporting information can be found online in the Supporting Information section.

## Figures and Tables

**TABLE 1 |:** Permanent dentition caries trajectories in the Dunedin Study birth cohort.^a^

Trajectory group	% Group membership^b^ (*n*)	Description
Group 1: Low caries rate	43.9% (431)	Low rates of untreated caries (DS), restored surfaces (FS), and teeth missing due to caries (MT).
Group 2: Moderate caries rate, maintained	24.6% (240)	Moderate caries experience rates (peak around ages 26 and 32 years), rising number of restored surfaces.
Group 3: Moderate caries rate, unmaintained	20.0% (194)	Moderate caries experience rates (peak around ages 26 and 32 years), fewer restored surfaces, higher tooth loss than previous group.
Group 4: High caries rate, restored	5.9% (57)	High caries rates and distinctly increasing number of restored surfaces.
Group 5: High caries rate, tooth loss	3.3% (31)	High caries rates and declining trajectories of DS and FS from age 32 years which corresponded with an increasing trajectory of missing teeth due to caries.
Group 6: High caries rate, untreated caries	2.2% (22)	High caries rates and an increasing trajectory of DS that might have not yet peaked by age 45 years.

a For a detailed description: [15].

b Proportion assigned to that group.

**TABLE 2 |:** Mental disorders at age 45 (Dunedin) and 40 (Christchurch) years by age 5/5–6 caries experience (dmft > 0).

	Dunedin Study				Christchurch Study			
	IRR	IRR 95% CI	*p*	*n*	IRR	IRR 95% CI	*p*	*n*
Mental disorders^a^								
Any internalizing disorder	1.13	0.90, 1.42	0.284	817	1.02	0.79, 1.31	0.909	817
Any externalizing disorder	0.93	0.73, 1.18	0.545	820	1.03	0.77, 1.39	0.824	817
Any thought disorder	1.22	0.67, 2.20	0.520	817	—	—	—	—
Generalized anxiety disorder	0.90	0.52, 1.57	0.717	816	0.94	0.35, 2.51	0.904	817
Any of six anxiety disorders	1.38	1.04, 1.85	0.028	816	1.03	0.69, 1.53	0.884	817
Major depression	0.75	0.55, 1.03	0.075	817	1.06	0.73, 1.55	0.746	817
Simple phobia	1.84	1.11, 3.04	0.018	797	1.39	0.77, 2.50	0.273	817
Social phobia	1.24	0.75, 2.04	0.399	801	1.09	0.52, 2.31	0.819	817
Alcohol dependence	1.04	0.71, 1.52	0.839	817	1.00	0.55, 1.80	0.987	817
Tobacco dependence	1.04	0.71, 1.52	0.842	816	1.09	0.76, 1.56	0.624	817
Conduct disorder	0.93	0.75, 1.15	0.509	817	—	—	—	—
Any mental disorder	1.01	0.86, 1.20	0.866	817	1.00	0.82, 1.21	0.965	817
Lifetime prevalence^b^	1.03	0.98, 1.08	0.305	875	0.99	0.93, 1.05	0.639	817

Abbreviations: CI = confidence interval; IRR = incidence rate ratio.

a Models adjusted for sex, childhood IQ, childhood SES, perinatal health, and adult personality. Comparison group = Caries-free (dmft = 0).

b In the Dunedin Study, comprised of any disorder from ages 11 to 45 years; in the Christchurch Study, comprised of any disorder from ages 14 to 40 years.

**TABLE 3 |:** Mental disorders at age 45 (Dunedin) and 40 (Christchurch) years by age 5/5–6 caries experience (dmft > 0).

	Dunedin Study				Christchurch Study			
	IRR	IRR 95% CI	*p*	*n*	IRR	IRR 95% CI	*p*	*n*
Mental disorders^a^								
Any internalizing disorder	1.13	0.90, 1.42	0.284	817	1.02	0.79, 1.31	0.909	817
Any externalizing disorder	0.93	0.73, 1.18	0.545	820	1.03	0.77, 1.39	0.824	817
Any thought disorder	1.22	0.67, 2.20	0.520	817	—	—	—	—
Generalized anxiety disorder	0.90	0.52, 1.57	0.717	816	0.94	0.35, 2.51	0.904	817
Any of six anxiety disorders	1.38	1.04, 1.85	0.028	816	1.03	0.69, 1.53	0.884	817
Major depression	0.75	0.55, 1.03	0.075	817	1.06	0.73, 1.55	0.746	817
Simple phobia	1.84	1.11, 3.04	0.018	797	1.39	0.77, 2.50	0.273	817
Social phobia	1.24	0.75, 2.04	0.399	801	1.09	0.52, 2.31	0.819	817
Alcohol dependence	1.04	0.71, 1.52	0.839	817	1.00	0.55, 1.80	0.987	817
Tobacco dependence	1.04	0.71, 1.52	0.842	816	1.09	0.76, 1.56	0.624	817
Conduct disorder	0.93	0.75, 1.15	0.509	817	—	—	—	—
Any mental disorder	1.01	0.86, 1.20	0.866	817	1.00	0.82, 1.21	0.965	817
Lifetime occurrence^b^	1.03	0.98, 1.08	0.305	875	0.99	0.93, 1.05	0.639	817

Abbreviations: CI = confidence interval; IRR = incidence rate ratio.

a Models adjusted for sex, childhood IQ, childhood SES, perinatal health, and adult personality. Comparison group = Caries-free (dmft = 0).

b In the Dunedin Study, comprised of any disorder from ages 11 to 45 years; in the Christchurch Study, comprised of any disorder from ages 14 to 40 years.

**TABLE 4 |:** Associations between permanent dentition caries trajectories and mental disorders among Dunedin Study participants at 45 years of age, models adjusted for sex, childhood SES and IQ, perinatal health, and adult personality.

	Any internalizing disorder (*n* = 893)		Any externalizing disorder (*n* = 896)		Any thought disorder (*n* = 893)		Any mental disorder (*n* = 893)		Lifetime prevalence (*n* = 944)	
	IRR (95% CI)	*p*	IRR (95% CI)	*p*	IRR (95% CI)	*p*	IRR (95% CI)	*p*	IRR (95% CI)	*p*
Permanent dentition caries trajectories										
Low caries rate	Ref.									
Moderate rate, maintained	1.00 (0.76, 1.30)	0.972	1.22 (0.87, 1.70)	0.254	0.95 (0.41, 2.17)	0.895	0.98 (0.79, 1.21)	0.865	1.07 (1.01, 1.14)	0.024
Moderate rate, unmaintained	0.98 (0.73, 1.32)	0.895	1.89 (1.39, 2.57)	< 0.001	1.80 (0.85, 3.80)	0.126	1.04 (0.84, 1.30)	0.719	1.09 (1.03, 1.16)	0.005
High rate, restored	1.09 (0.70, 1.70)	0.713	1.70 (1.05, 2.75)	0.031	1.47 (0.43, 4.97)	0.537	1.11 (0.79, 1.56)	0.556	1.05 (0.93, 1.17)	0.427
High rate, tooth loss	1.43 (1.00, 2.03)	0.050	2.58 (1.69, 3.93)	< 0.001	3.90 (1.43, 10.61)	0.008	1.36 (1.02, 1.80)	0.033	1.04 (0.95, 1.13)	0.444
High rate, untreated caries	1.16 (0.64, 2.12)	0.620	2.79 (1.78, 4.36)	< 0.001	5.02 (1.75, 14.40)	0.003	1.29 (0.89, 1.88)	0.175	1.12 (1.04, 1.21)	0.002
Sex										
Female	Ref.									
Male	0.65 (0.51, 0.81)	< 0.001	0.93 (0.73, 1.19)	0.555	0.67 (0.37, 1.21)	0.185	0.80 (0.67, 0.95)	0.012	0.91 (0.86, 0.96)	0.001
Childhood SES										
High	Ref.									
Medium	0.76 (0.56, 1.04)	0.088	0.70 (0.51, 0.97)	0.033	0.67 (0.31, 1.44)	0.309	0.72 (0.57, 0.91)	0.006	0.92 (0.86, 0.99)	0.021
Low	0.79 (0.54, 1.13)	0.199	0.79 (0.55, 1.15)	0.226	0.43 (0.18, 1.07)	0.069	0.72 (0.55, 0.95)	0.021	0.97 (0.90, 1.04)	0.376
Childhood IQ	0.99 (0.98, 1.00)	0.018	1.00 (0.99, 1.01)	0.461	0.99 (0.97, 1.01)	0.359	0.99 (0.99, 1.00)	0.006	1.00 (1.00, 1.00)	0.206
Personality										
Negative emotionality	1.38 (1.25, 1.53)	< 0.001	1.29 (1.16, 1.43)	< 0.001	1.76 (1.38, 2.25)	< 0.001	1.33 (1.23, 1.44)	< 0.001	1.08 (1.05, 1.10)	< 0.001
Positive emotionality	0.97 (0.88, 1.07)	0.567	1.02 (0.91, 1.13)	0.790	0.98 (0.76, 1.27)	0.902	0.98 (0.91, 1.06)	0.689	0.98 (0.96, 1.01)	0.145
Constraint	0.99 (0.88, 1.11)	0.832	0.79 (0.70, 0.89)	0.000	0.96 (0.73, 1.26)	0.765	0.90 (0.83, 0.98)	0.018	0.96 (0.93, 0.98)	0.001
Perinatal complications										
0	Ref.									
1+	1.10 (0.89, 1.35)	0.392	1.06 (0.85, 1.33)	0.586	1.01 (0.59, 1.72)	0.980	1.15 (0.98, 1.34)	0.092	0.99 (0.94, 1.04)	0.627

Abbreviations: CI = confidence interval; IRR = incidence rate ratio.

## Data Availability

The Dunedin Study data are available on request to the Study Director by qualified scientists. Requests require a concept paper describing the purpose of data access, ethical approval at the applicant’s institution, and provision for secure data access. We offer secure access to data on the Duke University, Otago University, and King’s College London campuses. All data analysis scripts and results files are available for review. The Christchurch Study data are available subject to the approval of the Study Director.

## References

[1] Wertz J, Caspi A, Ambler A, , “Association of History of Psychopathology With Accelerated Aging at Midlife,” JAMA Psychiatry 78, no. 5 (2021): 530–539. 33595619 10.1001/jamapsychiatry.2020.4626 PMC7890535

[2] World Health Organization, World Mental Health Report: Transforming Mental Health for all (World Health Organization, 2022), 296.

[3] Joury E, Kisely S, Watt RG, , “Mental Disorders and Oral Diseases: Future Research Directions,” Journal of Dental Research 102, no. 1 (2023): 5–12. 36081351 10.1177/00220345221120510

[4] World Health Organization, Mental Health Action Plan 2013–2020 (World Health Organization, 2013).

[5] Choi J, Price J, Ryder S, Siskind D, Solmi M, and Kisely S, “Prevalence of Dental Disorders Among People With Mental Illness: An Umbrella Review,” Australian and New Zealand Journal of Psychiatry 56, no. 8 (2022): 949–963. 34461748 10.1177/00048674211042239

[6] Delgado-Angulo EK, Sabbah W, Suominen AL, , “The Association of Depression and Anxiety With Dental Caries and Periodontal Disease Among Finnish Adults,” Community Dentistry and Oral Epidemiology 43, no. 6 (2015): 540–549. 26130047 10.1111/cdoe.12179

[7] Arora G, Humphris G, Lahti S, Richards D, and Freeman R, “Depression, Drugs and Dental Anxiety in Prisons: A Mediation Model Explaining Dental Decay Experience,” Community Dentistry and Oral Epidemiology 48, no. 3 (2020): 248–255. 32043284 10.1111/cdoe.12522

[8] Kisely S and Najman JM, “A Study of the Association Between Psychiatric Symptoms and Oral Health Outcomes in a Population-Based Birth Cohort at 30-Year-Old Follow-Up,” Journal of Psychosomatic Research 157 (2022): 110784. 10.1016/j.jpsychores.2022.110784 35325776

[9] Okoro CA, Strine TW, Eke PI, Dhingra SS, and Balluz LS, “The Association Between Depression and Anxiety and Use of Oral Health Services and Tooth Loss,” Community Dentistry and Oral Epidemiology 40, no. 2 (2012): 134–144. 10.1111/j.1600-0528.2011.00637.x 21883356

[10] Lopes AG, Ju X, Jamieson L, and Mialhe FL, “Oral Health-Related Quality of Life Among Brazilian Adults With Mental Disorders,” European Journal of Oral Sciences 129, no. 3 (2021): e12774. 10.1111/eos.12774 33786899

[11] Poulton R, Caspi A, Milne BJ, , “Association Between Children’s Experience of Socioeconomic Disadvantage and Adult Health: A Life-Course Study,” Lancet 360, no. 9346 (2002): 1640–1645. 12457787 10.1016/S0140-6736(02)11602-3 PMC3752775

[12] Anda RF, Felitti VJ, Bremner JD, , “The Enduring Effects of Abuse and Related Adverse Experiences in Childhood: A Convergence of Evidence From Neurobiology and Epidemiology,” European Archives of Psychiatry and Clinical Neuroscience 256, no. 3 (2006): 174–186. 16311898 10.1007/s00406-005-0624-4 PMC3232061

[13] Moffitt TE, Arseneault L, Belsky D, , “A Gradient of Childhood Self-Control Predicts Health, Wealth, and Public Safety,” Proceedings of the National Academy of Sciences of the United States of America 108, no. 7 (2011): 2693–2698. 21262822 10.1073/pnas.1010076108 PMC3041102

[14] Thomson WM, Broadbent JM, Caspi A, Poulton R, and Moffitt TE, “Childhood IQ Predicts Age-38 Oral Disease Experience and Service-Use,” Community Dentistry and Oral Epidemiology 47, no. 3 (2019): 252–258. 30812053 10.1111/cdoe.12451 PMC6520161

[15] Ruiz B, Broadbent JM, Thomson WM, Ramrakha S, Hong CL, and Poulton R, “Differential Unmet Needs and Experience of Restorative Dental Care in Trajectories of Dental Caries Experience: A Birth Cohort Study,” Caries Research 57, no. 4 (2023): 524–535. 37231938 10.1159/000530378

[16] Ruiz B, Broadbent JM, Thomson WM, , “Is Childhood Oral Health the ‘Canary in the Coal Mine’ for Poor Adult General Health? Findings From Two New Zealand Birth Cohort Studies,” Community Dentistry and Oral Epidemiology 51 (2023): 838–846. 36000812 10.1111/cdoe.12772

[17] Ruiz B, Broadbent JM, Thomson WM, , “Childhood Caries Is Associated With Poor Health and a Faster Pace of Aging by Midlife,” Journal of Public Health Dentistry 83, no. 4 (2023): 381–388. 37920118 10.1111/jphd.12591 PMC10919959

[18] Kisely S, Sawyer E, Siskind D, and Lalloo R, “The Oral Health of People With Anxiety and Depressive Disorders—A Systematic Review and Meta-Analysis,” Journal of Affective Disorders 200 (2016): 119–132. 27130961 10.1016/j.jad.2016.04.040

[19] Cademartori MG, Gastal MT, Nascimento GG, Demarco FF, and Corrêa MB, “Is Depression Associated With Oral Health Outcomes in Adults and Elders? A Systematic Review and Meta-Analysis,” Clinical Oral Investigations 22, no. 8 (2018): 2685–2702. 30191327 10.1007/s00784-018-2611-y

[20] Evans RW, Beck DJ, and Brown RH, “Dental Health of 5-Year-Old Children: A Report From the Dunedin Multidisciplinary Child Development Study,” New Zealand Dental Journal 76, no. 346 (1980): 179–186. 6935538

[21] Fergusson D and Horwood J, “Relationships Between Exposure to Additional Fluoride, Social Background and Dental Health in 7-Year-Old Children,” Community Dentistry and Oral Epidemiology 14, no. 1 (1986): 48–52. 3456874 10.1111/j.1600-0528.1986.tb01494.x

[22] Schaefer JD, Caspi A, Belsky DW, , “Enduring Mental Health: Prevalence and Prediction,” Journal of Abnormal Psychology 126, no. 2 (2017): 212–224. 27929304 10.1037/abn0000232 PMC5304549

[23] Melchior M, Moffitt TE, Milne BJ, Poulton R, and Caspi A, “Why Do Children From Socioeconomically Disadvantaged Families Suffer From Poor Health When They Reach Adulthood? A Life-Course Study,” American Journal of Epidemiology 166, no. 8 (2007): 966–974. 17641151 10.1093/aje/kwm155 PMC2491970

[24] Moffitt TE, Caspi A, Harrington H, , “Generalized Anxiety Disorder and Depression: Childhood Risk Factors in a Birth Cohort Followed to Age 32,” Psychological Medicine 37, no. 3 (2007): 441–452. 17201999 10.1017/S0033291706009640

[25] Anttila SS, Knuuttila MLE, and Sakki TK, “Relationship of Depressive Symptoms to Edentulousness, Dental Health, and Dental Health Behavior,” Acta Odontologica Scandinavica 59, no. 6 (2001): 406–412. 11831492 10.1080/000163501317153275

[26] Coles E, Chan K, Collins J, , “Decayed and Missing Teeth and Oral-Health-Related Factors: Predicting Depression in Homeless People,” Journal of Psychosomatic Research 71, no. 2 (2011): 108–112. 21767692 10.1016/j.jpsychores.2011.01.004

[27] Tyrovolas S, Koyanagi A, Panagiotakos DB, , “Population Prevalence of Edentulism and Its Association With Depression and Self-Rated Health,” Scientific Reports 6, no. 1 (2016): 37083. 27853193 10.1038/srep37083 PMC5112530

[28] Yamamoto T, Aida J, Kondo K, , “Oral Health and Incident Depressive Symptoms: JAGES Project Longitudinal Study in Older Japanese,” Journal of the American Geriatrics Society 65, no. 5 (2017): 1079–1084. 28165614 10.1111/jgs.14777

[29] Rouxel P, Tsakos G, Chandola T, and Watt RG, “Oral Health—A Neglected Aspect of Subjective Well-Being in Later Life,” Journals of Gerontology. Series B, Psychological Sciences and Social Sciences 73, no. 3 (2018): 382–386. 26970523 10.1093/geronb/gbw024 PMC5927002

[30] Zhang X, Hu X, Zhang Y, Sun J, and Chen G, “Longitudinal Association Between Oral Status and Depressive Symptoms Among Chinese Older Adults—China, 2014–2018,” China CDC Weekly 3, no. 40 (2021): 842–846. 34659865 10.46234/ccdcw2021.208 PMC8500802

[31] Matsuyama Y, Jürges H, Dewey M, and Listl S, “Causal Effect of Tooth Loss on Depression: Evidence From a Population-Wide Natural Experiment in the USA,” Epidemiology and Psychiatric Sciences 30 (2021): e38. 34030762 10.1017/S2045796021000287 PMC8157508

[32] Israel S, Moffitt TE, Belsky DW, , “Translating Personality Psychology to Help Personalize Preventive Medicine for Young Adult Patients,” Journal of Personality and Social Psychology 106, no. 3 (2014): 484–498. 24588093 10.1037/a0035687 PMC3951727

[33] Sukumaran I, Taylor S, and Thomson WM, “The Prevalence and Impact of Dental Anxiety Among Adult New Zealanders,” International Dental Journal 71, no. 2 (2020): 122–126. 32929752 10.1111/idj.12613 PMC9275063

[34] Benzian H, Daar A, and Naidoo S, “Redefining the Non-Communicable Disease Framework to a 6×6 Approach: Incorporating Oral Diseases and Sugars,” Lancet Public Health 8, no. 11 (2023): e899–e904. 37741288 10.1016/S2468-2667(23)00205-0

[35] Crocombe LA, Broadbent JM, Thomson WM, Brennan DS, Slade GD, and Poulton R, “Dental Visiting Trajectory Patterns and Their Antecedents,” Journal of Public Health Dentistry 71, no. 1 (2011): 23–31. 20880031 10.1111/j.1752-7325.2010.00196.x

