# Supplementary material for: Caries Trajectories From Childhood to Adulthood Associated With Mental Disorders in Midlife
Source: J Public Health Dent. Author manuscript; Available in PMC 2025 Jun 13. (PMC12147428; doi:10.1111/jphd.12665)
Supplement: sup table 1 [file NIHMS2077291-supplement-sup_table_1.docx]

Supplementary Table 1. Associations between highest quartile of dmfs/DMFS score at each age (from ages 5 to 45 years) and mental disorders at age 45 years among Dunedin Study participants.

|  | **Adjusted IRR^a^** | | | | | | | | | | | | | | | |
| --- | --- | --- | --- | --- | --- | --- | --- | --- | --- | --- | --- | --- | --- | --- | --- | --- |
|  | **Age 5 dmfs** | **IRR 95%CI** | **Age 9 DMFS** | **IRR 95%CI** | **Age 15 DMFS** | **IRR 95%CI** | **Age 18 DMFS** | **IRR 95%CI** | **Age 26 DMFS** | **IRR 95%CI** | **Age 32 DMFS** | **IRR 95%CI** | **Age 38 DMFS** | **IRR 95%CI** | **Age 45 DMFS** | **IRR 95%CI** |
| **Mental disorders** | |  |  |  |  |  |  |  |  |  |  |  |  |  |  |  |
| Any internalising disorder | 0.77 | 0.59, 1.01 | 0.98 | 0.72, 1.33 | 0.93 | 0.71, 1.22 | 1.14 | 0.90, 1.44 | 1.05 | 0.83, 1.34 | 1.06 | 0.84, 1.35 | 0.99 | 0.78, 1.24 | 1.13 | 0.90, 1.41 |
| Any externalising disorder | 0.97 | 0.74, 1.27 | 0.93 | 0.66, 1.31 | 0.95 | 0.71, 1.28 | 0.99 | 0.75, 1.32 | 1.27 | 0.99, 1.62 | 1.47 | 1.16, 1.87 | 1.43 | 1.12, 1.82 | 1.49 | 1.17, 1.89 |
| Any thought disorder | 1.10 | 0.58, 2.10 | 0.63 | 0.22, 1.79 | 1.29 | 0.68, 2.46 | 1.06 | 0.55, 2.06 | 1.43 | 0.77, 2.64 | 1.44 | 0.78, 2.66 | 1.66 | 0.96, 2.88 | 2.15 | 1.25, 3.68 |
| Generalised anxiety disorder | 0.54 | 0.24, 1.19 | 0.77 | 0.30, 1.98 | 1.43 | 0.77, 2.66 | 1.52 | 0.87, 2.65 | 0.87 | 0.46, 1.65 | 1.27 | 0.71, 2.28 | 1.05 | 0.59, 1.86 | 1.30 | 0.73, 2.32 |
| Any of 6 anxiety disorders | 0.95 | 0.69, 1.29 | 0.98 | 0.67, 1.45 | 1.29 | 0.95, 1.74 | 1.44 | 1.09, 1.89 | 1.33 | 1.00, 1.77 | 1.43 | 1.08, 1.90 | 1.15 | 0.87, 1.52 | 1.27 | 0.96, 1.68 |
| Major depression | 0.67 | 0.45, 1.01 | 0.82 | 0.51, 1.34 | 0.65 | 0.41, 1.01 | 0.85 | 0.57, 1.26 | 0.67 | 0.44, 1.01 | 0.85 | 0.58, 1.23 | 1.01 | 0.72, 1.41 | 1.07 | 0.76, 1.49 |
| Simple phobia | 1.27 | 0.78, 2.06 | 1.08 | 0.56, 2.11 | 1.40 | 0.84, 2.34 | 1.95 | 1.24, 3.06 | 1.65 | 1.03, 2.62 | 1.31 | 0.81, 2.11 | 0.92 | 0.57, 1.49 | 1.04 | 0.65, 1.67 |
| Social phobia | 0.51 | 0.26, 1.01 | 0.97 | 0.50, 1.91 | 0.82 | 0.45, 1.48 | 0.80 | 0.46, 1.41 | 0.62 | 0.33, 1.14 | 0.68 | 0.38, 1.22 | 0.66 | 0.38, 1.15 | 0.67 | 0.38, 1.17 |
| Alcohol dependence | 1.13 | 0.74, 1.73 | 0.89 | 0.51, 1.55 | 0.69 | 0.40, 1.19 | 0.74 | 0.45, 1.23 | 0.92 | 0.59, 1.43 | 0.95 | 0.61, 1.48 | 1.05 | 0.69, 1.60 | 0.86 | 0.56, 1.34 |
| Tobacco dependence | 0.98 | 0.63, 1.52 | 1.22 | 0.75, 1.98 | 1.22 | 0.76, 1.95 | 1.31 | 0.82, 2.07 | 1.70 | 1.16, 2.48 | 2.17 | 1.47, 3.20 | 2.03 | 1.37, 3.02 | 2.05 | 1.39, 3.03 |
| Conduct disorder | 1.08 | 0.85, 1.37 | 1.07 | 0.80, 1.44 | 1.05 | 0.82, 1.36 | 1.12 | 0.87, 1.43 | 1.22 | 0.97, 1.54 | 1.25 | 0.99, 1.58 | 1.11 | 0.88, 1.40 | 1.20 | 0.96, 1.50 |
| Any mental disorder | 0.85 | 0.70, 1.05 | 0.99 | 0.78, 1.25 | 0.93 | 0.76, 1.14 | 1.03 | 0.85, 1.24 | 1.14 | 0.96, 1.36 | 1.12 | 0.94, 1.34 | 1.05 | 0.88, 1.25 | 1.14 | 0.96, 1.35 |
| Lifetime occurrence ^b^ | 1.00 | 0.94, 1.05 | 1.02 | 0.95, 1.09 | 1.03 | 0.98, 1.09 | 1.04 | 0.99, 1.10 | 1.03 | 0.98, 1.08 | 1.02 | 0.97, 1.08 | 1.02 | 0.97, 1.08 | 1.03 | 0.97, 1.08 |

^a^Models adjusted for sex, childhood IQ, childhood SES, perinatal health, and adult personality. Comparison group = caries experience in lower quartiles (Q1-Q3). ^b^In the Dunedin Study, comprised of any diagnosis from ages 11 to 45 years. Abbreviations: IRR = incidence rate ratio, CI = confidence interval.
