# Supplementary material for: Caries Trajectories From Childhood to Adulthood Associated With Mental Disorders in Midlife
Source: J Public Health Dent. Author manuscript; Available in PMC 2025 Jun 13. (PMC12147428; doi:10.1111/jphd.12665)
Supplement: sup table 2 [file NIHMS2077291-supplement-sup_table_2.docx]

**Analyses without controlling for adult personality:**

Supplementary Table 2. Mental disorders at age 45 (Dunedin) and 40 (Christchurch) years by age 5/5-6 caries experience (dmft>0), (without controlling for adult personality).

|  | **Dunedin Study** | | | | **Christchurch Study** | | | |
| --- | --- | --- | --- | --- | --- | --- | --- | --- |
|  | **IRR** | **IRR 95% CI** | **p** | **n** | **IRR** | **IRR 95% CI** | **p** | **n** |
| **Mental disorders^a^** |  |  |  |  |  |  |  |  |
| Any internalising disorder | 1.13 | 0.90, 1.43 | 0.284 | 827 | 0.98 | 0.75, 1.27 | 0.865 | 822 |
| Any externalising disorder | 0.93 | 0.73, 1.18 | 0.533 | 830 | 1.02 | 0.76, 1.38 | 0.873 | 818 |
| Any thought disorder | 1.24 | 0.69, 2.23 | 0.470 | 827 | - | - | - | - |
| Generalised anxiety disorder | 0.94 | 0.55, 1.62 | 0.828 | 826 | 0.91 | 0.36, 2.29 | 0.839 | 822 |
| Any of 6 anxiety disorders | 1.40 | 1.04, 1.87 | 0.025 | 826 | 1.01 | 0.67, 1.51 | 0.976 | 822 |
| Major depression | 0.76 | 0.55, 1.05 | 0.099 | 827 | 1.00 | 0.68, 1.46 | 0.998 | 822 |
| Simple phobia | 1.81 | 1.09, 3.00 | 0.021 | 807 | 1.37 | 0.74, 2.51 | 0.316 | 822 |
| Social phobia | 1.20 | 0.72, 1.99 | 0.479 | 811 | 0.96 | 0.46, 1.98 | 0.904 | 822 |
| Alcohol dependence | 1.03 | 0.71, 1.52 | 0.861 | 827 | 0.96 | 0.54, 1.71 | 0.885 | 818 |
| Tobacco dependence | 1.02 | 0.69, 1.51 | 0.923 | 826 | 1.09 | 0.76, 1.56 | 0.630 | 818 |
| Conduct disorder | 0.91 | 0.73, 1.13 | 0.383 | 827 | - | - | - | - |
| Any mental disorder | 1.01 | 0.85, 1.21 | 0.886 | 827 | 0.98 | 0.81, 1.20 | 0.872 | 822 |
| Lifetime prevalence^b^ | 1.02 | 0.96, 1.07 | 0.560 | 908 | 0.99 | 0.93, 1.05 | 0.664 | 967 |

^a^Models adjusted for sex, childhood IQ, childhood SES, perinatal health. Comparison group = Caries-free (dmft=0). ^b^In the Dunedin Study, comprised of any disorder from ages 11 to 45 years; in the Christchurch Study, comprised of any disorder from ages 14 to 40 years. Abbreviations: IRR = incidence rate ratio, CI = confidence interval.
