# Supplementary material for: Caries Trajectories From Childhood to Adulthood Associated With Mental Disorders in Midlife
Source: J Public Health Dent. Author manuscript; Available in PMC 2025 Jun 13. (PMC12147428; doi:10.1111/jphd.12665)
Supplement: sup table 3 [file NIHMS2077291-supplement-sup_table_3.docx]

Supplementary Table 3. Associations between permanent dentition caries trajectories and mental disorders among Dunedin Study participants at 45 years of age, models adjusted for sex, childhood SES and IQ, and perinatal health (without controlling for adult personality).

|  | **Any  internalising  disorder (n=900)** |  | **Any externalising  disorder (n=903)** |  | **Any  thought  disorder  (n=900)** |  | **Any  mental disorder (n=900)** |  | **Lifetime prevalence (n=959)** |  |
| --- | --- | --- | --- | --- | --- | --- | --- | --- | --- | --- |
|  | **IRR (95% CI)** | **p** | **IRR (95% CI)** | **p** | **IRR (95% CI)** | **p** | **IRR (95% CI)** | **p** | **IRR (95% CI)** | **p** |
| **Permanent dentition caries trajectories** | | | |  |  |  |  |  |  |  |
| Low caries rate | Ref. | | |  | | | | | | |
| Moderate rate, maintained | 1.01 (0.77, 1.34) | 0.923 | 1.24 (0.88, 1.75) | 0.217 | 0.98 (0.42, 2.32) | 0.971 | 1.00 (0.80, 1.25) | 0.996 | 1.08 (1.01, 1.15) | 0.021 |
| Moderate rate, unmaintained | 1.13 (0.85, 1.51) | 0.398 | 2.26 (1.67, 3.05) | 0.000 | 2.41(1.21, 4.80) | 0.012 | 1.22 (0.98, 1.52) | 0.078 | 1.14 (1.08, 1.21) | <0.001 |
| High rate, restored | 1.07 (0.69, 1.67) | 0.767 | 1.65 (1.02, 2.67) | 0.042 | 1.39 (0.41, 4.68) | 0.594 | 1.08 (0.77, 1.53) | 0.651 | 1.04 (0.93, 1.17) | 0.471 |
| High rate, tooth loss | 1.91 (1.33, 2.75) | 0.001 | 3.68 (2.47, 5.50) | <0.001 | 6.15 (2.53, 14.91) | <0.001 | 1.87 (1.41, 2.49) | <0.001 | 1.15 (1.05, 1.25) | 0.002 |
| High rate, untreated caries | 1.54 (0.85, 2.78) | 0.153 | 3.91(2.63, 5.81) | <0.001 | 7.55 (2.97, 19.19) | <0.001 | 1.74 (1.21, 2.51) | 0.003 | 1.22 (1.15, 1.30) | <0.001 |
| **Sex** | | | |  | | | | | | |
| Female | Ref. | | |  |  |  |  |  |  |  |
| Male | 0.71 (0.57, 0.88) | 0.002 | 1.20 (0.95, 1.51) | 0.130 | 0.85 (0.50, 1.45) | 0.558 | 0.93 (0.79, 1.09) | 0.375 | 0.95 (0.91, 1.00) | 0.055 |
| **Childhood SES** | | | |  | | | | | | |
| High | Ref. | | |  |  |  |  |  |  |  |
| Medium | 0.81 (0.60, 1.10) | 0.178 | 1.20 (0.95, 1.51) | 0.130 | 0.73 (0.34, 1.54) | 0.406 | 0.76 (0.60, 0.95) | 0.018 | 0.93 (0.87, 1.00) | 0.049 |
| Low | 0.87 (0.61, 1.25) | 0.451 | 0.85 (0.59, 1.23) | 0.399 | 0.57 (0.24, 1.36) | 0.207 | 0.79 (0.60, 1.04) | 0.089 | 0.99 (0.92, 1.07) | 0.876 |
| **Childhood IQ** | 0.99 (0.98, 1.00) | 0.002 | 1.00 (0.99, 1.01) | 0.570 | 0.99 (0.96, 1.01) | 0.157 | 0.99 (0.98, 1.00) | 0.002 | 1.00 (1.00, 1.00) | 0.157 |
| **Perinatal complications** | | | |  | | | | | | |
| 0 | Ref. | | | | | | | | | |
| 1+ | 1.13 (0.92, 1.39) | 0.250 | 1.09 (0.87, 1.36) | 0.458 | 1.10 (0.66, 1.85) | 0.707 | 1.18 (1.00, 1.38) | 0.049 | 0.99 (0.94, 1.04) | 0.633 |

Abbreviations: IRR = incidence rate ratio, CI = confidence interval.
