# Supplementary material for: Caries Trajectories From Childhood to Adulthood Associated With Mental Disorders in Midlife
Source: J Public Health Dent. Author manuscript; Available in PMC 2025 Jun 13. (PMC12147428; doi:10.1111/jphd.12665)
Supplement: sup table 4 [file NIHMS2077291-supplement-sup_table_4.docx]

**Supporting Information**

**Caries trajectories from childhood to adulthood associated with mental disorders in midlife**

Begoña Ruiz, Jonathan M. Broadbent, W. Murray Thomson, Sandhya Ramrakha, Joseph Boden, Geri McLeod, Terrie E. Moffitt, Avshalom Caspi, Richie Poulton.

**Study design and description of cohorts**

The Dunedin Study is a population-representative birth cohort of 1037 individuals (91% of eligible births; 52% boys) born from 1 April 1972 to 31 March 1973 in Dunedin, NZ (Poulton et al. 2015). Cohort families represent the full SES range of NZ’s South Island, and study participants are primarily of European ethnicity (7.5% self-identify as Māori, and 1.5% as Pacific people). Perinatal data were collected at birth, and the cohort for the longitudinal study was defined at age 3 years. The cohort has been assessed at ages 5, 7, 9, 11, 13, 15, 18, 21, 26, 32, 38, and (most recently) at age 45 years, when 938 (94%) of the 997 living cohort members took part. Written informed consent and ethical approval were gained for each assessment.

The Christchurch Study is a birth cohort of 1265 individuals (50.2% boys) born from 15 April to 5 August 1977 in Christchurch, NZ. The cohort comprises 97% of all births occurring during that period (Fergusson and Horwood 2001). Cohort families represent the full SES range of NZ’s South Island. Most participants self-identify as being of European origin, but about 13% report Māori or Paciﬁc ethnicity. The cohort has been assessed at birth, 4 months, annually from age 1 to 16 years and again at ages 18, 21, 25, 30, 35 and 40 years, when 904 (74%) of the 1222 living cohort members participated. Written informed consent and ethical approval were gained for each assessment.

**Explanatory variables: Assessing dental caries experience**

In the Dunedin Study, as previously reported (Broadbent et al. 2008; Broadbent et al. 2013; Thomson et al. 2013), dental examinations for caries and missing teeth at each age (5, 9, 15, 18, 26, 32, 38 and 45 years) were conducted using calibrated dental examiners. Dental examiners were not aware of study members’ SES or questionnaire responses at the time of the examinations. Details on the oral health examinations at each assessment age are reported elsewhere (Ruiz et al. 2023). Teeth were examined for caries and restorations following WHO methods (World Health Organization 1977), with 4 surfaces being considered for anterior teeth (canines and incisors): and 5 surfaces for posterior teeth (premolars and molars). Only those teeth that had been lost because of caries are included in estimations of tooth loss due to caries and in the ‘M’ component of DMF scores. Teeth extracted for reasons other than dental caries (such as impaction or orthodontic treatment) were not included in the computation of tooth loss.

In the Christchurch Study, the first phase of dental data collection was conducted in 1982-1983. Clinical dental data was obtained from the routinely-collected records of the first and seconds visits made to a School Dental Service (SDS) clinic at ages 5 or 6 years. The examinations were undertaken according to the clinical judgement of the examiners (dental nurses). The SDS records accounted for the number of visits the child had made to the school dental clinic every year, accordingly, each child could have data from multiple examinations each year, but only the first visits were reported here. Children’s caries experience was summarised using the dmft index (Klein et al. 1938). No dental-surface data were available in the Christchurch Study.

Permanent dentition caries trajectories (Dunedin cohort)

Group-based multi-trajectory modelling was used to identify developmental trajectories of untreated carious tooth surfaces (DS), restored tooth surfaces (FS), and teeth extracted due to caries (MT) from ages 9 to 45 years in a New Zealand longitudinal birth cohort, the Dunedin Multidisciplinary Health and Development Study (n=975). The selected trajectory model included six trajectory groups, with trajectories following a cubic function for DS, a quadratic function for FS, and a linear function for MT.

Six trajectory groups were identified and labelled: ‘low caries rate’; ‘moderate caries rate, maintained’; ‘moderate caries rate, unmaintained’; ‘high caries rate, restored’; ‘high caries rate, tooth loss’; and ‘high caries rate, untreated caries’.

Briefly, the first group accounted for 43.9% of the cohort (n = 431), and was labeled “low caries rate” group (with low untreated caries, restored surfaces, and teeth missing due to caries). The second and third groups were similar in their (moderate) caries rates, but differed in the number of fillings, whereby in the “moderate caries rate, maintained” group (24.6% of participants, n = 240) there was a rising number of restored surfaces that roughly doubled the count of FS in the “moderate caries rate, unmaintained” group (20.0%, n = 194). These groups were labeled “maintained” and “unmaintained,” to reflect their differential experience of restorative dental care. The last three groups had high caries rates; but differed in the relative proportion of dental restorations (FS), extractions (MT), or remained untreated (DS). The fourth group (5.9%, n = 57), labeled “high caries rate, restored,” is characterized by a distinctly increasing number of restored surfaces. The fifth group (3.2%, n = 31), labeled “high caries rate, tooth loss,” showed declining trajectories of DS and FS from age 32 years which corresponded with an increasing trajectory of missing teeth due to caries. The last group (2.2%, n = 22) labeled “high caries rate, untreated caries” showed an increasing trajectory of DS that might have not yet peaked by age 45 years. More details on the permanent dentition caries trajectories from ages 9 through to 45 years have been reported elsewhere (Ruiz et al. 2023).

**Outcome variables: Mental disorders in midlife**

In the Dunedin Study, mental disorders have been assessed at every assessment phase since age 11 years, and most recently at age 45 years (Caspi et al. 2020). At age 45 years, psychiatric interviews were conducted by health professionals blind to participants’ prior data, using the Diagnostic Interview Schedule (Robins et al. 1981; Robins et al. 1995). The participants were asked about past-year symptoms based on the ‘Diagnostic and Statistical Manual of Mental Disorders’ (DSM) criteria. The following disorders were assessed: externalising disorders (attention-deficit/hyperactivity disorder, conduct disorder, alcohol dependence, tobacco dependence, cannabis dependence, and drug dependence); internalising disorders (major depression, generalised anxiety disorder (GAD), fears (including social phobia, specific/simple phobia, agoraphobia, and panic disorder), posttraumatic stress disorder (PTSD) and eating disorders (including bulimia and anorexia); and thought disorders (obsessive-compulsive disorder (OCD), mania, and schizophrenia). Composite measures of ‘any internalising disorder’, ‘any externalising disorder’, ‘any thought disorder’, and ‘any mental disorder at age 45 years’ were created in the present analyses. Additionally, a measure of ‘any lifetime mental disorder by age 45 years’ (lifetime occurrence) was used. For the latter, data on mental disorders at ages 11, 13, 15, 18, 21, 26, 32, 38 and 45 years were used.

At age 15 years, interviews used the Diagnostic Interview Schedule for Children (Costello et al. 1982) and disorders were assessed according to DSM-III (American Psychiatric Association 1980). From ages 18 to 45 years, the interviews used the Diagnostic Interview Schedule (Robins et al. 1981; Robins et al. 1995). At ages 18 and 21 years, disorders were assessed using DMS-III revised (American Psychiatric Association 1987); at ages 26, 32, and 38 years, disorders were assessed using DSM-IV (American Psychiatric Association 1994); and at age 45 years, DSM-5 (fifth edition) was used (American Psychiatric Association 2013). The exception was substance-dependence disorders, which were diagnosed at age 45 using DSM-IV because the dependence and abuse distinction had been removed from DSM-5.

In the Christchurch Study at age 40 years, as previously described (McLeod et al. 2022), study members completed a detailed mental health interview where they were asked about past-year symptoms of mental and substance use using the Composite International Diagnostic Interview (CIDI) (World Health Organization 1993). Custom written survey items were used to assess DSM-5 diagnostic criteria for the following disorders: externalising disorders (alcohol dependence, cannabis dependence, drug dependence (not cannabis), tobacco dependence); internalising disorders (major depression, generalised anxiety disorder (GAD), social phobia, specific/simple phobia, agoraphobia, and panic disorder), posttraumatic stress disorder (PTSD), eating disorders (including bulimia, anorexia and binge eating disorder), and manic episode. Composite measures of ‘any internalising disorder’, and ‘any externalising disorder’, and ‘any mental disorder at age 40 years’ were used. A composite measure of ‘any lifetime mental disorder by age 40 years’ (lifetime prevalence) used data on mental disorders from ages 15-16, 18, 21, 25, 30, 35, and 39-40 years.

At each assessment from age 15 to age 40 years, mental health interviews were conducted to assess DSM symptom criteria. At the age 15-16 assessment, study members and their parents were asked separately about the participant’s mental health in the previous year using a mix of standardised assessment tools including the Diagnostic Interview for Children (Costello et al. 1982), the Diagnostic Interview Schedule (Robins et al. 1995), the Rutgers Alcohol Problems Index (Raskin White and Labouvie 1989), together with custom written survey items to assess DMS-III revised (American Psychiatric Association 1987) symptom criteria. Participants were classified as having a disorder if they met diagnostic criteria on the basis of either self- or parent-report, since this method was shown to provide the optimal method of classification (Fergusson et al. 1996). From ages 18 to 35 years, participants were assessed with the Composite International Diagnostic Interview (CIDI) (World Health Organization 1993) and custom written survey items to assess DSM-IV symptom criteria (American Psychiatric Association 1994). The data were combined over all assessments to construct a lifetime measure (from ages 15 to 40 years) reflecting whether the participant had ever met diagnostic criteria for any disorder.

**Covariates**

The selection of covariates was guided by previous knowledge of some of the psychosocial and widers determinants of oral and mental health. These covariates are known determinants of oral and mental health, have been previously published, and have good reliability and validity in this cohort. All analyses adjusted for sex, perinatal health, childhood SES, childhood IQ and adult personality. Analyses were guided by a directed acyclic graph (Figure S1).

**
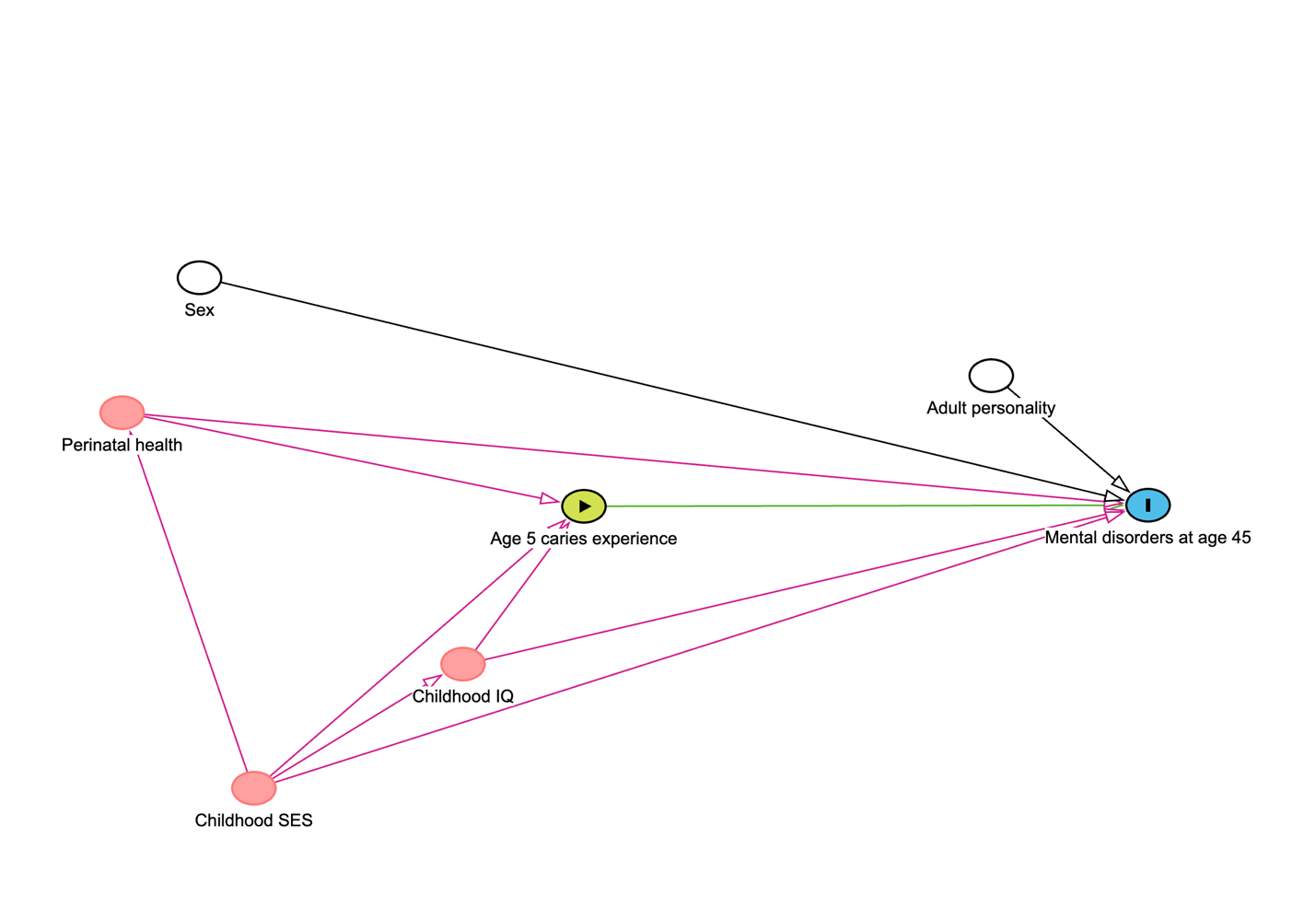
**

**Figure S1.** Supplementary figure S1. Directed acyclic graph conceptualising the analytical approach.

Childhood socioeconomic status (SES): Childhood socioeconomic background (SES) reflects the socioeconomic conditions experienced by the participants while they were growing up. This measure was used as a covariate because the social, economic and environmental conditions in which individuals and populations grow, live and work throughout the lifecourse greatly influence health (Ben-Shlomo and Kuh 2002; Marmot 2010). In both studies, family SES was recorded at the child’s birth based on the Elley-Irving scale of socioeconomic status for New Zealand, which places occupations into one of six categories ranging from 1=professional to 6=unskilled labourer (Elley and Irving 1976). In the Christchurch study, scale scores were usually based on the nature of the father’s employment. In the Dunedin study, the average of the highest occupation level of either parent assessed repeatedly for each participant at 3, 5, 7, 9, 11, and 15 years of age was used.

Childhood IQ: Childhood IQ was used as a covariate because childhood cognitive ability is associated with health and longevity, health behaviours, ageing (Schaefer et al. 2016) and mortality (Calvin et al. 2011; Sörberg Wallin et al. 2018). Childhood cognitive function is also a key determinant of oral health and dental service-use by midlife (Thomson et al. 2019). In the Dunedin study, childhood IQ was assessed using the Wechsler Intelligence Scale for Children–Revised (WISC–R) (Wechsler 1974) which was administered to the participants at ages 7, 9, and 11 years. The tests were individually administered at each assessment according to standard protocol. The IQ variable used for these analyses was the averaged measure of IQs determined at these three ages, standardised to population norms with a mean of 100 and a standard deviation of 15 (Caspi et al. 2020). In the Christchurch study, IQ was also assessed using the same scale at age 8-9 years, standardised to a mean of 100 and a standard deviation of 15 (Spittlehouse et al. 2020).

Personality style: Personality traits are associated with positive mental health and psychopathology. Healthy personality development contributes to many areas of wellbeing (Kang et al. 2023). At age 26 years, participants in the Dunedin study completed a 177-item version of the Multidimensional Personality Questionnaire (MPQ) adapted for New Zealand (Caspi et al. 2003). The MPQ personality scales define ten lower-order level aspects (subscales) of personality and three higher-order level superfactors: negative emotionality, positive emotionality, and constraint. As previously described (Slutske et al. 2005), individuals scoring high on the dimension of positive emotionality have a lower threshold for the experience of positive emotions and for positive engagement in their social and work environments, and they tend to view life as being essentially a pleasurable experience. Low scorers report fear of these pleasurable transactions, a low degree of self-efficacy (the belief that they can influence their environment) and are less likely to be happy (Thomson et al. 2011). The positive emotionality scale is a combination of scores from the lower-order MPQ scales of wellbeing, social potency, achievement, and social closeness. Individuals scoring high on the dimension of negative emotionality have a low general threshold for the experience of negative emotions such as anxiety and anger, and they tend to breakdown under stress. The negative emotionality scale is a combination of scores from the lower-order MPQ scales of stress reaction, alienation, and aggression. Individuals scoring high on the dimension of constraint tend to endorse conventional social norms, avoid thrills, and act in a cautious and restrained manner, while low scorers are impulsive, fearless, and sensation-seeking, and reject conventional strictures on their behaviour (Thomson et al. 2011). The constraint scale is a combination of scores from the lower-order MPQ scales of self-control, harm avoidance, and traditionalism. For the purpose of the present analyses, the continuous MPQ superfactor scale scores were standardised into Z scores so that the mean and SD for each scale were 0 and 1, respectively.

At age 40 years, Christchurch study members’ personality style was assessed according to Goldberg’s ‘Big-Five’ broad-bandwidth dimensions of personality (Goldberg 1990). The ‘Big-Five’ refer to five independent dimensions of personality often referred to as: Extraversion, Agreeableness, Conscientiousness, Neuroticism, and Openness to Experience. As previously described (Sibley et al. 2011; Sibley and Pirie 2013), individuals scoring high on Extraversion are those who engage in social endeavours and their example traits are sociability, leadership and exhibition. Individuals scoring high on Agreeableness are described as having in-group co-operation and tolerance, their example traits are tolerance, forgiveness, (low) quarrelsomeness. Individuals scoring high on Conscientiousness are those engaging in task-related endeavours, their example traits are diligence, organisation and attention to detail. Example traits of individuals scoring high on Neuroticism (low emotional stability) are anxiety, insecurity and (low) calmness. Individuals scoring high on Openness to Experience are engaged in ideas-related endeavours and their example traits are curiosity, imaginativeness, (low) need for cognitive closure and (low) need for certainty. For the purpose of the present analyses, the continuous scores of Extraversion, Conscientiousness, and Neuroticism scales were standardised into Z scores so that the mean and SD for each were 0 and 1, respectively, to match the analogue personality traits in the Dunedin study.

Perinatal complications: Perinatal health was used as a covariate because extensive research has shown that adverse conditions during *in-utero* growth (early development) have an impact on future chronic disease experience and early mortality. In the Dunedin study, as previously described (Shalev et al. 2014) each child was examined shortly after birth, and perinatal information was taken from the hospital records. The obstetric complications assessed in this study—including prenatal, intrapartum, and neonatal complications—were maternal diabetes, glycosuria, epilepsy, hypertension, eclampsia, antepartum hemorrhage, accidental hemorrhage, placenta previa, having had a previous small baby, gestational age <37 weeks or >41 weeks, birth weight <2.5 kg, small size for gestational age, major or minor neurologic signs of the neonatal period (eg, jitteriness, tenseness, limpness, hypotonicity), Rh incompatibility, ABO incompatibility, non-hemolytic hyperbilirubinemia, hypoxia at birth (idiopathic respiratory distress syndrome or apnea), and low Apgar score at birth. The infant was defined as having a low Apgar score if 1 of the following conditions applied: (a) at 5 minutes of life, the infant’s heart rate was 100 beats per minute, respiration was irregular or absent, and the infant was centrally cyanotic; (b) the infant took >10 minutes to establish normal respiration; or (c) the infant’s asphyxia at birth warranted resuscitation. The sum of maternal complications and neonate complications was significantly and positively correlated (r = 0.156, P< 0.001). Based on evidence that the effects of adverse conditions are cumulative, each condition was weighted equally and summed to yield an obstetric complications index. This was then recoded so that Study members were scored as 0, 1, 2+ when having none, 1, or ≥2 perinatal complications, respectively.

In the Christchurch study, perinatal complications assessed included: mother had previous low birthweight infant, antepartum haemorrhage, maternal high (diastolic) blood pressure, pre-eclamptic toxaemia, gestational age <37 weeks or >41 weeks, birth weight <2.5 kg, high bilirubin level, significant Rh or other blood incompatibility accompanied by high bilirubin, hypoxia or respiratory distress at birth, and low Apgar score ≤8 at 5 minutes. The perinatal complications index was computed as the sum of the above indicators, accordingly each study member was classified as 0, 1, 2+ when having none, 1, or ≥2 perinatal complications, respectively. For the present analyses, in both studies, Study members were categorised as 0 with no, or as 1+ where there were ≥1 perinatal complications.

**Supplementary Table 1.** Associations between highest quartile of dmfs/DMFS score at each age (from ages 5 to 45 years) and mental disorders at age 45 years among Dunedin Study participants.

|  | **Adjusted IRR^a^** | | | | | | | | | | | | | | | |
| --- | --- | --- | --- | --- | --- | --- | --- | --- | --- | --- | --- | --- | --- | --- | --- | --- |
|  | **Age 5 dmfs** | **IRR 95%CI** | **Age 9 DMFS** | **IRR 95%CI** | **Age 15 DMFS** | **IRR 95%CI** | **Age 18 DMFS** | **IRR 95%CI** | **Age 26 DMFS** | **IRR 95%CI** | **Age 32 DMFS** | **IRR 95%CI** | **Age 38 DMFS** | **IRR 95%CI** | **Age 45 DMFS** | **IRR 95%CI** |
| **Mental disorders** | |  |  |  |  |  |  |  |  |  |  |  |  |  |  |  |
| Any internalising disorder | 0.77 | 0.59, 1.01 | 0.98 | 0.72, 1.33 | 0.93 | 0.71, 1.22 | 1.14 | 0.90, 1.44 | 1.05 | 0.83, 1.34 | 1.06 | 0.84, 1.35 | 0.99 | 0.78, 1.24 | 1.13 | 0.90, 1.41 |
| Any externalising disorder | 0.97 | 0.74, 1.27 | 0.93 | 0.66, 1.31 | 0.95 | 0.71, 1.28 | 0.99 | 0.75, 1.32 | 1.27 | 0.99, 1.62 | 1.47 | 1.16, 1.87 | 1.43 | 1.12, 1.82 | 1.49 | 1.17, 1.89 |
| Any thought disorder | 1.10 | 0.58, 2.10 | 0.63 | 0.22, 1.79 | 1.29 | 0.68, 2.46 | 1.06 | 0.55, 2.06 | 1.43 | 0.77, 2.64 | 1.44 | 0.78, 2.66 | 1.66 | 0.96, 2.88 | 2.15 | 1.25, 3.68 |
| Generalised anxiety disorder | 0.54 | 0.24, 1.19 | 0.77 | 0.30, 1.98 | 1.43 | 0.77, 2.66 | 1.52 | 0.87, 2.65 | 0.87 | 0.46, 1.65 | 1.27 | 0.71, 2.28 | 1.05 | 0.59, 1.86 | 1.30 | 0.73, 2.32 |
| Any of 6 anxiety disorders | 0.95 | 0.69, 1.29 | 0.98 | 0.67, 1.45 | 1.29 | 0.95, 1.74 | 1.44 | 1.09, 1.89 | 1.33 | 1.00, 1.77 | 1.43 | 1.08, 1.90 | 1.15 | 0.87, 1.52 | 1.27 | 0.96, 1.68 |
| Major depression | 0.67 | 0.45, 1.01 | 0.82 | 0.51, 1.34 | 0.65 | 0.41, 1.01 | 0.85 | 0.57, 1.26 | 0.67 | 0.44, 1.01 | 0.85 | 0.58, 1.23 | 1.01 | 0.72, 1.41 | 1.07 | 0.76, 1.49 |
| Simple phobia | 1.27 | 0.78, 2.06 | 1.08 | 0.56, 2.11 | 1.40 | 0.84, 2.34 | 1.95 | 1.24, 3.06 | 1.65 | 1.03, 2.62 | 1.31 | 0.81, 2.11 | 0.92 | 0.57, 1.49 | 1.04 | 0.65, 1.67 |
| Social phobia | 0.51 | 0.26, 1.01 | 0.97 | 0.50, 1.91 | 0.82 | 0.45, 1.48 | 0.80 | 0.46, 1.41 | 0.62 | 0.33, 1.14 | 0.68 | 0.38, 1.22 | 0.66 | 0.38, 1.15 | 0.67 | 0.38, 1.17 |
| Alcohol dependence | 1.13 | 0.74, 1.73 | 0.89 | 0.51, 1.55 | 0.69 | 0.40, 1.19 | 0.74 | 0.45, 1.23 | 0.92 | 0.59, 1.43 | 0.95 | 0.61, 1.48 | 1.05 | 0.69, 1.60 | 0.86 | 0.56, 1.34 |
| Tobacco dependence | 0.98 | 0.63, 1.52 | 1.22 | 0.75, 1.98 | 1.22 | 0.76, 1.95 | 1.31 | 0.82, 2.07 | 1.70 | 1.16, 2.48 | 2.17 | 1.47, 3.20 | 2.03 | 1.37, 3.02 | 2.05 | 1.39, 3.03 |
| Conduct disorder | 1.08 | 0.85, 1.37 | 1.07 | 0.80, 1.44 | 1.05 | 0.82, 1.36 | 1.12 | 0.87, 1.43 | 1.22 | 0.97, 1.54 | 1.25 | 0.99, 1.58 | 1.11 | 0.88, 1.40 | 1.20 | 0.96, 1.50 |
| Any mental disorder | 0.85 | 0.70, 1.05 | 0.99 | 0.78, 1.25 | 0.93 | 0.76, 1.14 | 1.03 | 0.85, 1.24 | 1.14 | 0.96, 1.36 | 1.12 | 0.94, 1.34 | 1.05 | 0.88, 1.25 | 1.14 | 0.96, 1.35 |
| Lifetime occurrence ^b^ | 1.00 | 0.94, 1.05 | 1.02 | 0.95, 1.09 | 1.03 | 0.98, 1.09 | 1.04 | 0.99, 1.10 | 1.03 | 0.98, 1.08 | 1.02 | 0.97, 1.08 | 1.02 | 0.97, 1.08 | 1.03 | 0.97, 1.08 |

^a^Models adjusted for sex, childhood IQ, childhood SES, perinatal health, and adult personality. Comparison group = caries experience in lower quartiles (Q1-Q3). ^b^In the Dunedin Study, comprised of any diagnosis from ages 11 to 45 years. Abbreviations: IRR = incidence rate ratio, CI = confidence interval.

Analyses without controlling for adult personality:

**Supplementary Table 2.** Mental disorders at age 45 (Dunedin) and 40 (Christchurch) years by age 5/5-6 caries experience (dmft>0), (without controlling for adult personality).

|  | **Dunedin Study** | | | | **Christchurch Study** | | | |
| --- | --- | --- | --- | --- | --- | --- | --- | --- |
|  | **IRR** | **IRR 95% CI** | **p** | **n** | **IRR** | **IRR 95% CI** | **p** | **n** |
| **Mental disorders^a^** |  |  |  |  |  |  |  |  |
| Any internalising disorder | 1.13 | 0.90, 1.43 | 0.284 | 827 | 0.98 | 0.75, 1.27 | 0.865 | 822 |
| Any externalising disorder | 0.93 | 0.73, 1.18 | 0.533 | 830 | 1.02 | 0.76, 1.38 | 0.873 | 818 |
| Any thought disorder | 1.24 | 0.69, 2.23 | 0.470 | 827 | - | - | - | - |
| Generalised anxiety disorder | 0.94 | 0.55, 1.62 | 0.828 | 826 | 0.91 | 0.36, 2.29 | 0.839 | 822 |
| Any of 6 anxiety disorders | 1.40 | 1.04, 1.87 | 0.025 | 826 | 1.01 | 0.67, 1.51 | 0.976 | 822 |
| Major depression | 0.76 | 0.55, 1.05 | 0.099 | 827 | 1.00 | 0.68, 1.46 | 0.998 | 822 |
| Simple phobia | 1.81 | 1.09, 3.00 | 0.021 | 807 | 1.37 | 0.74, 2.51 | 0.316 | 822 |
| Social phobia | 1.20 | 0.72, 1.99 | 0.479 | 811 | 0.96 | 0.46, 1.98 | 0.904 | 822 |
| Alcohol dependence | 1.03 | 0.71, 1.52 | 0.861 | 827 | 0.96 | 0.54, 1.71 | 0.885 | 818 |
| Tobacco dependence | 1.02 | 0.69, 1.51 | 0.923 | 826 | 1.09 | 0.76, 1.56 | 0.630 | 818 |
| Conduct disorder | 0.91 | 0.73, 1.13 | 0.383 | 827 | - | - | - | - |
| Any mental disorder | 1.01 | 0.85, 1.21 | 0.886 | 827 | 0.98 | 0.81, 1.20 | 0.872 | 822 |
| Lifetime prevalence^b^ | 1.02 | 0.96, 1.07 | 0.560 | 908 | 0.99 | 0.93, 1.05 | 0.664 | 967 |

^a^Models adjusted for sex, childhood IQ, childhood SES, perinatal health. Comparison group = Caries-free (dmft=0). ^b^In the Dunedin Study, comprised of any disorder from ages 11 to 45 years; in the Christchurch Study, comprised of any disorder from ages 14 to 40 years. Abbreviations: IRR = incidence rate ratio, CI = confidence interval.

**Supplementary Table 3.** Associations between permanent dentition caries trajectories and mental disorders among Dunedin Study participants at 45 years of age, models adjusted for sex, childhood SES and IQ, and perinatal health (without controlling for adult personality).

|  | **Any  internalising  disorder (n=900)** |  | **Any externalising  disorder (n=903)** |  | **Any  thought  disorder  (n=900)** |  | **Any  mental disorder (n=900)** |  | **Lifetime prevalence (n=959)** |  |
| --- | --- | --- | --- | --- | --- | --- | --- | --- | --- | --- |
|  | **IRR (95% CI)** | **p** | **IRR (95% CI)** | **p** | **IRR (95% CI)** | **p** | **IRR (95% CI)** | **p** | **IRR (95% CI)** | **p** |
| **Permanent dentition caries trajectories** | | | |  |  |  |  |  |  |  |
| Low caries rate | Ref. | | |  | | | | | | |
| Moderate rate, maintained | 1.01 (0.77, 1.34) | 0.923 | 1.24 (0.88, 1.75) | 0.217 | 0.98 (0.42, 2.32) | 0.971 | 1.00 (0.80, 1.25) | 0.996 | 1.08 (1.01, 1.15) | 0.021 |
| Moderate rate, unmaintained | 1.13 (0.85, 1.51) | 0.398 | 2.26 (1.67, 3.05) | 0.000 | 2.41(1.21, 4.80) | 0.012 | 1.22 (0.98, 1.52) | 0.078 | 1.14 (1.08, 1.21) | <0.001 |
| High rate, restored | 1.07 (0.69, 1.67) | 0.767 | 1.65 (1.02, 2.67) | 0.042 | 1.39 (0.41, 4.68) | 0.594 | 1.08 (0.77, 1.53) | 0.651 | 1.04 (0.93, 1.17) | 0.471 |
| High rate, tooth loss | 1.91 (1.33, 2.75) | 0.001 | 3.68 (2.47, 5.50) | <0.001 | 6.15 (2.53, 14.91) | <0.001 | 1.87 (1.41, 2.49) | <0.001 | 1.15 (1.05, 1.25) | 0.002 |
| High rate, untreated caries | 1.54 (0.85, 2.78) | 0.153 | 3.91(2.63, 5.81) | <0.001 | 7.55 (2.97, 19.19) | <0.001 | 1.74 (1.21, 2.51) | 0.003 | 1.22 (1.15, 1.30) | <0.001 |
| **Sex** | | | |  | | | | | | |
| Female | Ref. | | |  |  |  |  |  |  |  |
| Male | 0.71 (0.57, 0.88) | 0.002 | 1.20 (0.95, 1.51) | 0.130 | 0.85 (0.50, 1.45) | 0.558 | 0.93 (0.79, 1.09) | 0.375 | 0.95 (0.91, 1.00) | 0.055 |
| **Childhood SES** | | | |  | | | | | | |
| High | Ref. | | |  |  |  |  |  |  |  |
| Medium | 0.81 (0.60, 1.10) | 0.178 | 1.20 (0.95, 1.51) | 0.130 | 0.73 (0.34, 1.54) | 0.406 | 0.76 (0.60, 0.95) | 0.018 | 0.93 (0.87, 1.00) | 0.049 |
| Low | 0.87 (0.61, 1.25) | 0.451 | 0.85 (0.59, 1.23) | 0.399 | 0.57 (0.24, 1.36) | 0.207 | 0.79 (0.60, 1.04) | 0.089 | 0.99 (0.92, 1.07) | 0.876 |
| **Childhood IQ** | 0.99 (0.98, 1.00) | 0.002 | 1.00 (0.99, 1.01) | 0.570 | 0.99 (0.96, 1.01) | 0.157 | 0.99 (0.98, 1.00) | 0.002 | 1.00 (1.00, 1.00) | 0.157 |
| **Perinatal complications** | | | |  | | | | | | |
| 0 | Ref. | | | | | | | | | |
| 1+ | 1.13 (0.92, 1.39) | 0.250 | 1.09 (0.87, 1.36) | 0.458 | 1.10 (0.66, 1.85) | 0.707 | 1.18 (1.00, 1.38) | 0.049 | 0.99 (0.94, 1.04) | 0.633 |

Abbreviations: IRR = incidence rate ratio, CI = confidence interval.

**Supporting information - References**

American Psychiatric Association. 1980. Diagnostic and statistical manual of mental disorders. 3rd ed. Washington, D.C: American Psychiatric Association.

American Psychiatric Association. 1987. Diagnostic and statistical manual of mental disorders. 3rd ed, re. Washington, D.C: American Psychiatric Association.

American Psychiatric Association. 1994. Diagnostic and statistical manual of mental disorders. 4th ed. Washington, D.C: American Psychiatric Association.

American Psychiatric Association. 2013. Diagnostic and statistical manual of mental disorders: DSM-5. 5th ed. Washington, D.C: American Psychiatric Association.

Ben-Shlomo Y, Kuh D. 2002. A life course approach to chronic disease epidemiology: Conceptual models, empirical challenges and interdisciplinary perspectives. International Journal of Epidemiology. 31(2):285–293.

Broadbent JM, Foster Page LA, Thomson WM, Poulton R. 2013. Permanent dentition caries through the first half of life. Br Dent J. 215(7):1–6.

Broadbent JM, Thomson WM, Poulton R. 2008. Trajectory patterns of dental caries experience in the permanent dentition to the fourth decade of life. J Dent Res. 87(1):69–72.

Calvin CM, Deary IJ, Fenton C, Roberts BA, Der G, Leckenby N, Batty GD. 2011. Intelligence in youth and all-cause-mortality: Systematic review with meta-analysis. International journal of epidemiology. 40(3):626–644.

Caspi A, Harrington H, Milne B, James W. Amell, Theodore R, Moffitt TE. 2003. Children’s behavioral styles at age 3 are linked to their adult personality traits at age 26. J Pers. 71(4):495–514.

Caspi A, Houts RM, Ambler A, Danese A, Elliott ML, Hariri A, Harrington HL, Hogan S, Poulton R, Ramrakha S, et al. 2020. Longitudinal assessment of mental health disorders and comorbidities across 4 decades among participants in the Dunedin birth cohort study. JAMA Netw Open. 3(4):1–14.

Costello A, Edelbrock C, Kalas R, Kessler M, Klaric S. 1982. Diagnostic interview schedule for children (DISC). Bethesda, MD: National Institute of Mental Health.

Elley WB, Irving JC. 1976. Revised socio-economic index for New Zealand. New Zeal J Educ Stud. 11:25–36.

Fergusson DM, Horwood LJ. 2001. The Christchurch health and development study: Review of findings on child and adolescent mental health. Aust N Z J Psychiatry. 35(3):287–296.

Fergusson DM, Lynskey MT, Horwood LJ. 1996. Comorbidity between depressive disorders and nicotine dependence in a cohort of 16-year-olds. Arch Gen Psychiatry. 53(11):1043–1047.

Goldberg LR. 1990. An alternative “Description of Personality”: The Big-Five factor structure. J Pers Soc Psychol. 59(6):1216–1229.

Kang W, Steffens F, Pineda S, Widuch K, Malvaso A. 2023. Personality traits and dimensions of mental health. Sci Rep. 13(1):1–10.

Klein H, Palmer CE, Knutson JW. 1938. Studies on dental caries: I. Dental status and dental needs of elementary school children. Public Health Reports (1896-1970). 53(19):751–765.

Marmot M. 2010. Fair Society, Healthy Lives: The Marmot Review; Strategic Review of Health Inequalities in England post-2010. London: Institute of Health Equity.

McLeod GFH, Cleland L, Welch J, Spittlehouse JK, Fenton A, Boden JM, Horwood LJ. 2022. Menopause status and climacteric symptoms in a birth cohort of mid-life New Zealand women. Climacteric. 25(3):271–277.

Poulton R, Moffitt TE, Silva PA. 2015. The Dunedin multidisciplinary health and development study: Overview of the first 40 years, with an eye to the future. Soc Psychiatry Psychiatr Epidemiol. 50(5):679–693.

Raskin White H, Labouvie EW. 1989. Towards the assessment of adolescent problem drinking. J Stud Alcohol. 50(1):30–37.

Robins LN, Cottler L, Bucholz K, Compton W. 1995. Diagnostic Interview Schedule for DSM-IV. St. Louis, MO: Washington University Press.

Robins LN, Helzer JE, Croughan J, Ratcliff KS. 1981. National Institute of Mental Health Diagnostic Interview Schedule: Its history, characteristics, and validity. Arch Gen Psychiatry. 38(4):381–389.

Schaefer JD, Caspi A, Belsky DW, Harrington H, Houts R, Israel S, Levine ME, Sugden K, Williams B, Poulton R, et al. 2016. Early-Life Intelligence Predicts Midlife Biological Age. The journals of gerontology Series B, Psychological sciences and social sciences. 71(6):968–977.

Shalev I, Caspi A, Ambler A, Belsky DW, Chapple S, Cohen HJ, Israel S, Poulton R, Ramrakha S, Rivera CD, et al. 2014. Perinatal complications and aging indicators by midlife. Pediatrics. 134(5):e1315–e1323.

Sibley C, Luyten N, Wolfman M, Mobberley A, Wootton LW, Hammond M, Sengupta N, Perry R, West-Newman T, Wilson M, et al. 2011. The Mini-IPIP6: Validation and extension of a short measure of the Big-Six factors of personality in New Zealand. NZ J Psychol. 40:142–159.

Sibley C, Pirie DJ. 2013. Personality in New Zealand: Scale norms and demographic differences in the mini-IPIP6. NZ J Psychol. 42:13–30.

Slutske WS, Caspi A, Moffitt TE, Poulton R. 2005. Personality and problem gambling: A prospective study of a birth cohort of young adults. Arch Gen Psychiatry. 62(7):769–775.

Sörberg Wallin A, Allebeck P, Gustafsson JE, Hemmingsson T. 2018. Childhood IQ and mortality during 53 years’ follow-up of Swedish men and women. Journal of Epidemiology and Community Health. 72(10):926–932.

Spittlehouse JK, Boden JM, Horwood LJ. 2020. Sexual orientation and mental health over the life course in a birth cohort. Psychol Med. 50(8):1348–1355.

Thomson WM, Broadbent JM, Caspi A, Poulton R, Moffitt TE. 2019. Childhood IQ predicts age-38 oral disease experience and service-use. Community dentistry and oral epidemiology. 47(3):252–258.

Thomson WM, Broadbent JM, Foster Page LA, Poulton R. 2013. Antecedents and associations of root surface caries experience among 38-year-olds. Caries Res. 47(2):128–134.

Thomson WM, Caspi A, Poulton R, Moffitt TE, Broadbent JM. 2011. Personality and oral health. Eur J Oral Sci. 119(5):366–372.

Wechsler D. 1974. Manual for the Wechsler intelligence scale for children - Revised. San Antonio, TX: The Psychological Corporation.

World Health Organization. 1977. Oral health surveys: Basic methods. Second ed. Geneva: World Health Organization.

World Health Organization. 1993. Composite International Diagnostic Interview (CIDI). Geneva: World Health Organization.
